# First case of penicillin-resistant *Streptococcus agalactiae* (group B *Streptococcus*) isolated from an adult patient in Italy, February 2026

**DOI:** 10.2807/1560-7917.ES.2026.31.20.2600374

**Published:** 2026-05-21

**Authors:** Roberta Creti, Giovanni Gherardi, Monica Imperi, Andrea Zappavigna, Gabriella Tocci, Nicolò Rossi, Filippo Trapani, Giovanna Alfarone, Ilaria Marani, Anna Teresa Palamara, Giuliana Lo Cascio

**Affiliations:** 1 Department of Infectious Diseases, Istituto Superiore di Sanità, Rome, Italy; 2 Department of Laboratory Medicine, Microbiology Unit- AUSL, Piacenza, Italy; 3 Department of Specialistic Medicine, Infectious Disease Unit- AUSL, Piacenza, Italy; 4 Department of Medicine and Surgery- University of Parma, Parma, Italy

**Keywords:** *Streptococcus agalactiae*, group B streptococci, penicillin-resistance, *pbp2x*, *pbp2a*, multidrug-resistance

## Abstract

A penicillin-resistant group B streptococcal (GBS) strain was identified in Italy, after isolation from the blood culture of a patient in their 80s with comorbidities. The isolate was multidrug-resistant and demonstrated by genome sequencing to be of serotype Ia, sequence type 23, and to possess the *alp1* gene. Known and new point mutations were detected in the penicillin-binding-protein genes 2a and 2x. Although sporadic, penicillin-resistance in GBS is a serious concern for prophylaxis and therapy, so continued GBS surveillance remains important.

Penicillin is the first-line agent for both prophylaxis and treatment of *Streptococcus agalactiae* (also known as group B *Streptococcus*, GBS) infections [1]. According to the European Committee on Antimicrobial Susceptibility Testing (EUCAST) 2026 guideline [2], the epidemiological cut-off (ECOFF) for penicillin in GBS is 0.125 mg/L. While isolates of GBS are considered universally susceptible to penicillin, strains with penicillin minimum inhibitory concentration (MIC) values above the ECOFF (penicillin-resistant GBS, PRGBS) that exhibit point mutations in the penicillin-binding protein (PBP) genes *pbp*2x, *pbp*1a, *pbp*2a and *pbp*2b are increasingly reported [3]. Here we present the microbiological characterisation of a multidrug-resistant (MDR) PRGBS strain identified in Italy, reporting its clonal origin and the mutations detected in *pbp* genes, which were possibly involved in the resistance phenotype.

## Case description and treatment

In early 2026, a patient aged in their early 80s with a history of mitral stenosis and recent pacemaker implantation presented to the emergency department with fever (≥ 38.5 °C), lower back pain, and signs of heart failure. These symptoms had begun on the previous day. The patient was admitted to the cardiology ward, and empirical antibiotic therapy with ceftriaxone was started after blood cultures were initiated. Preliminary blood culture results revealed oxacillin-susceptible *Staphylococcus aureus* and *Streptococcus* spp.; therefore, antimicrobial therapy was de-escalated to oxacillin. Definitive microbiological identification confirmed the presence of *Staphylococcus aureus* resistant only to penicillin and *Streptococcus agalactiae* exhibiting resistance to penicillin, clindamycin, tetracycline, and levofloxacin. Based on these findings, antimicrobial therapy was escalated to vancomycin. Follow-up blood cultures performed 48 hours and 96 hours after the treatment onset were negative, and transthoracic echocardiography showed no evidence of infective endocarditis. Due to contraindications to magnetic resonance imaging related to the presence of the pacemaker, a fluorine‑18 fluorodeoxyglucose positron emission tomography/computerised tomography (^18^F-FDG PET/CT) scan was performed 1 week after hospital admission, demonstrating increased metabolic uptake at the lumbosacral joint level, consistent with spondylodiscitis. Antimicrobial therapy was subsequently switched to rifampicin and trimethoprim/sulfamethoxazole, and the patient was discharged in stable clinical condition. 

## Microbiological analysis of the *Streptococcus agalactiae* strain

From a microbiological perspective, the *Streptococcus agalactiae* strain was isolated from blood cultures collected in a BioMérieux BacT/Alert FA Plus bottle, which turned positive after 10.9 hours of incubation. Subcultures were performed on agar plates, and species identification was achieved using matrix-assisted laser desorption/ionisation – time of flight (MALDI-TOF) mass spectrometry (Vitek-MS system). Antimicrobial susceptibility testing was performed using the semi-automated Vitek 2 system and E-test, showing penicillin MICs of 0.25 mg/L and 0.19 mg/L, respectively, consistent with penicillin resistance according to both the EUCAST ECOFF and clinical breakpoint. Other antibiotic susceptibilities were interpreted using the EUCAST clinical breakpoints [4]. The GBS isolate resulted susceptible to vancomycin, teicoplanin, linezolid, rifampicin, and trimethoprim/sulfamethoxazole, but appeared to be MDR [5] as it showed resistance to levofloxacin (MIC > 8 mg/L; breakpoint: 2 mg/L), erythromycin (MIC = 2 mg/L; breakpoint: 0.25 mg/L), and tetracycline (MIC = 4 mg/L; breakpoint: 1 mg/L).

The isolate was sent for further testing to the National Reference Laboratory for β-haemolytic streptococci at the Istituto Superiore di Sanità (ISS), Rome where it was registered as strain ISS-11151. Broth microdilution method was performed using Sensititre plates (ThermoFisher Scientific) according to the manufacturer’s instructions. Results confirmed that the isolate was PRGBS with a MIC for penicillin of 0.25 mg/L, and MDR, with resistance to erythromycin (MIC = 8 mg/L), levofloxacin (MIC > 4 mg/L), tetracycline (MIC > 8 mg/L) and inducible resistance to clindamycin. The isolate was tested for high-level gentamicin resistance, and it was susceptible showing a MIC value < 128 mg/L and the absence of the *aac(6′)-aph(2)″* gene [6].

## Molecular investigation

Whole genome sequencing was performed at the ISS using MinIon (Oxford Nanopore Technologies), according to the manufacturer’s instruction. High-quality reads were de novo assembled. Genome assembly was annotated using PGAP v2025–05–06.build7983.

Molecular typing was performed by submitting the genome sequence to PubMLST (https://pubmlst.org/) that identified the ISS-11151 strain as serotype Ia, sequence type (ST) 23 (clonal complex 23) and possessing the alpha-like protein 1 gene (*alp*1), a surface protein acting as an important virulence factor and used in vaccine formulations under development.

Deduced amino acid sequences of PBP2a, PBP2b, PBP2x and PBP1a proteins from GBS ISS-11151 genome were compared with those of the penicillin-susceptible GBS reference isolates A909 (Ia, ST7; GenBank accession number: CP000114.1) and 515 (Ia, ST23; GenBank accession number: CP051004).

The substitution E156V in the protein PBP2a and three substitutions, namely H401L, L437I, H438Y in the PBP2x protein were peculiar of the penicillin-resistant GBS-11151 strain compared with the penicillin-susceptible reference GBS strains (Table).

**Table:** Identification of amino acid differences between the PBP proteins of the PRGBS investigated here and those of penicillin susceptible GBS A909 and 515 strains of same serotype and clonal complex, Italy, 2026 (n = 3 strains)

PRGBS strain ID	Encoded proteins	Mutations encoded by 11151 compared to peptide sequence of reference strains	
		A909 (Ia, ST7) reference strain	515 (Ia, ST23) reference strain
11151	PBP1a	A27T; V744A; G739S; N740del; N741del	No difference
	PBP2a	K63E; **E156V**	**E156V**
	PBP2x^a^	I342V (I377V); **H366L (H401L)**; **L402I (L437I)**; H403Y **(H438Y)**; V475I (V510I)	**H401L; L437I; H438Y**
	PBP2b	S192N	No difference

PBP: penicillin-binding protein; PRGBS: penicillin-resistant group B *Streptococcus.*

^a^ The A909 annotated pbp2X (SAK_0359) in the GenBank entry lacks the first 35 amino acids so that the amino acid positions are shifted. In parenthesis the real positions are reported.

The amino acid substitutions that are peculiar only to the PRGBS strain investigated here are in bold.

## Discussion

Group B *Streptococcus* infections have been documented in humans and animals [7]. In humans, although GBS is a commensal of the gastrointestinal and genitourinary tracts, it may lead to invasive disease in very young (i.e. neonates − including pre-term, and infants) or older (aged ≥ 65 years) individuals [1]. While GBS resistance to penicillin, the first-line agent for both prophylaxis and treatment, remains rare, increasing numbers of PRGBS reports worldwide [3] have contributed, alongside other criteria, to include PRGBS in the 2024 World Health Organization (WHO) Bacterial Priority Pathogens List [8].

Here we report to the best of our knowledge the first case of invasive GBS infection caused by a PRGBS strain in Italy, where the surveillance of invasive GBS infections in humans is conducted on a voluntary basis [9,10]. The strain was additionally MDR, as has been frequently observed in previous descriptions of PRGBS [11,12]. Unlike our case, however, most prior reported PRGBS strains were recovered from older adults with chronic prosthetic joint infections who received long-term antibiotic treatments [3,13-15]. These events highlighted the possible risk of point mutations accumulation driven by the long-term antibiotic selection pressure.

The PRGBS isolate reported here was of serotype Ia/ST23. Other studies find PRGBS serotype Ia, but without specifying the genetic lineage [16,17] or involve different serotypes/lineages [11,15,18]. Serotype Ia/ST23 PRGBS are reported from the United States (US) and Germany [12,14,19], but the mutations in the *pbp*2X and *pbp*2a genes that are considered important for the resistance phenotype in these investigations are different from those detected in our PRGBS isolate. Other than serotype Ia, a recent review indicated also serotype III to be mostly associated with PRGBS isolates [3].

Indeed, phylogenetic studies have indicated that multiple PRGBS genetic lineages arose independently, due to the accumulation of mutations, particularly in the *pbp2x* gene [12,16,19-21]. The comparison of different serotype/clonal lineages of PRGBS can, however, bias the identification of the specific substitutions in the *pbp* genes that confer the penicillin resistance phenotype. For this reason, we focused our analysis on the phylogenetically closest reference genomes which allowed us to identify only a few amino acid substitutions in the PBP2a and PBP2x proteins. 

Previous publications have considered the amino acid changes V405A and Q557E in the *pbp2x* gene as key for the development of PRGBS because they are close to the conserved active site [3]. Nevertheless, the Q557E mutation has been also reported in penicillin-susceptible GBS, suggesting that this mutation may represent a common first step in the development of penicillin resistance [22]. V405A and Q557E mutations in PBP2X were not observed in our PRGBS isolate, while they were present in the PRGBS Ia/ST23 from Germany [14], but not in the US PRGBS strains [12].

Three PBP2X mutations (H401L, L437I and H438Y) were considered peculiar of our PRGBS because they were absent in the two GBS reference strains considered. Of note, the mutation H438Y has been previously reported in both PRGBS and penicillin-susceptible isolates [14,23,24], suggesting that this point mutation may contribute, but not be essential to the resistant-conferring phenotype. The other two mutations H401L and L437I, to the best of our knowledge, have not previously been reported in PBP2X of other PRGBS isolates. Interestingly, H401L is very close to the conserved transpeptidase domain active site. Furthermore, we identified the mutation E156V in the PBP2A which has also not been described. The PBP1A and PBP2B protein sequences were identical to the reference strain 515.

The current work presented has a few limitations. First, while we refer to GBS isolates with MICs higher than the ECOFF as PRGBS, this is a conservative definition, as the term ‘resistance’ may not be sufficiently robust for this species. Indeed, there is a paucity of available clinical data on the therapeutic success or failure of high-dose antibiotic regimens when considering MIC values slightly above the ECOFF. Moreover, in a few studies, the acquisition of PBP mutations by GBS isolates recovered from patients undergoing β-lactam therapy has not been associated with treatment failure [13,25]. Second, to find the most relevant mutations underlying the penicillin-resistant phenotype of our isolate, we only compared its PBP amino acid sequences to those from the phylogenetically closest reference strains; however, this does not exclude the possibility that additional mutations not detected with this approach could act as a scaffold for the substitutions we identified.

## Conclusion

Here, we describe the first case of invasive infection caused by a PRGBS strain in Italy, which was also MDR. While the clinical relevance of our PRGBS isolate remains uncertain, the impact of its detection in terms of antimicrobial resistance is clear. Because PRGBS strains, as in our case, are frequently MDR, drugs should be carefully chosen for a clinically effective treatment. Our results moreover highlight the importance of continued surveillance of antibiotic resistance in GBS infections in the country, which is corroborated by the inclusion of PRGBS in the WHO Bacterial Priority Pathogens List, 2024. From a One Health perspective, GBS also causes infections in animals so appropriate antibiotic use within specific stewardship programmes is fundamental in all different sectors to avoid the emergence of difficult to treat GBS infections.

## Data Availability

The assembled GBS-11151 genome has been submitted to Genbank with the accession number JBYAXE000000000.
